# Supplementary material for: Study on Quality Control of Compound Anoectochilus roxburghii (Wall.) Lindl. by Liquid Chromatography–Tandem Mass Spectrometry
Source: Molecules. 2022 Jun 27;27(13):4130. doi: 10.3390/molecules27134130 (PMC9268268; doi:10.3390/molecules27134130)
Supplement: Supplementary file 1 [file molecules-27-04130-s001.zip › molecules-1787154-supplementary.pdf]

Tables Caption

Table S1. Recoveries of eight analytes in CAROL

Table S2. Recoveries of the extract of nucleosides

Table S3. Recoveries of the extract of ganoderic acid

Table S1. Recoveries of eight analytes in CAROL

Analytes	Original (μg)	Spiked (μg)	Detected (μg)	Recovery (%)	Mean recovery (%)	RSD % (n=6)
	1.21	0.90	2.04	91.73		
Adenine	1.12	1.50	2.53	93.69	92.41	1.31
	1.07	2.10	2.99	91.79		
	7.72	5.00	12.26	90.94		
Uridine	7.33	8.40	14.78	88.69	90.05	2.49
	7.44	10.10	16.58	90.53		
	0.09	0.05	0.13	95.32		
2'-Deoxyuridine	0.09	0.08	0.16	90.37	92.36	2.93
	0.08	0.11	0.18	91.40		
	0.0111	0.0060	0.0168	94.83		
2'-Deoxyadeno-sine	0.0117	0.0100	0.0214	96.63	96.86	3.08
	0.0102	0.0140	0.0241	99.13		
	6.57	4.20	10.50	93.57		
Guanosine	6.61	7.00	12.84	89.00	90.09	3.31
	6.30	9.80	14.89	87.71		
	2.10	1.38	3.32	88.03		
Lucidenic acid A	2.00	2.30	3.92	83.14	85.07	3.04
	1.77	3.22	4.48	84.03		
	19.29	13.80	33.11	100.16		
Ganoderic acid F	18.67	23.00	40.72	95.90	97.50	2.41
	18.13	32.20	49.19	96.46		
	39.14	21.00	57.41	86.96		
Ganoderic acid A	36.79	35.00	67.48	87.66	86.42	1.72
	34.36	49.00	75.83	84.63		

Table S2. Recoveries of the extract of nucleosides

Analytes	*A. roxburghii* form Mingxi					*G. lucidum* form Longyang				
	**Original (μg)**	**Spiked (μg)**	**Detected (μg)**	**Mean recovery (%)**	**RSD % (n=3)**	**Original (μg)**	**Spiked (μg)**	**Detected (μg)**	**Mean recovery (%)**	**RSD % (n=3)**
Adenine	0.01	0.25	0.23	89.37	0.30	0.09	0.25	0.30	84.25	0.97
Uridine	6.42	10.00	14.68	82.65	0.52	3.67	5.00	8.61	90.38	1.00
2'-Deoxyuridine	0.07	0.05	0.12	86.08	0.74	0.02	0.05	0.06	84.49	0.93
2'-Deoxyadenosine	0.92	1.00	1.90	97.61	1.10	0.02	0.05	0.07	94.27	0.77
Guanosine	2.31	2.50	4.40	83.77	0.78	1.68	2.50	3.66	84.21	0.31

Table S3. Recoveries of the extract of ganoderic acid

Analytes	water extract of *G. lucidum*					ethanol extract of *G. lucidum*				
	**Original (μg)**	**Spiked (μg)**	**Detected (μg)**	**Mean recovery (%)**	**RSD % (n=3)**	**Original (μg)**	**Spiked (μg)**	**Detected (μg)**	**Mean recovery (%)**	**RSD % (n=3)**
Lucidenic acid A	1.30	3.20	3.92	81.86	0.73	1.29	3.20	4.01	85.06	0.36
Ganoderic acid F	8.54	9.60	16.75	85.54	0.22	8.15	9.60	16.21	84.02	0.78
Ganoderic acid A	26.22	16.00	40.47	89.08	0.90	26.95	16.00	40.56	85.08	0.48
